# Clinical Remarks upon Surgical Cases in the Buffalo Hospital of the Sisters of Charity

**Published:** 1873-11

**Authors:** J. F. Miner


ART. II.—*Clinical Remarks upon Surgical Cases in the Buffalo Hospital of the Sisters of Charity.* By Prof. J. F. MINER, M. D

Reported by E. N. BRUSH.

Saturday, Oct., 11th.—Gentlemen: The case which I bring before you this morning as an introductory to our clinical study in this hospital is that of a young man, J. B———, aged 24, who is suffering with stricture of the Urethra. He is sent to us by a very capable physician from out of town, with the following history. Somewhat over two years ago he contracted gonorrhœa, for which he received treatment, a few months after he noticed that his urine was voided in a smaller stream than usual. The difficulty in passing his water has gradually increased until he is now only able to urinate a drop at a time. Stricture of the urethra is as you know, qualified by several terms as: idiopathic, traumatic, congenital, passable, impassable, spasmodic, etc., of the signification of these terms it is unnecessary to speak, as they sufficiently explain themselves.

Stricture when idiopathic is as a general rule the result of inflammation of the urethra. Of the pathology of stricture, time will not permit me to speak, save to say, that as the result of the inflammation of the urethra, a plastic material is thrown out in the sub-mucous cellular tissue, which diminishes the size of the urethra; in time this plastic material becoming organized, contracts, becomes hard and dense, similar to cicatrices in other localities, resulting in true organic stricture.

His physician has made attempts to introduce a catheter, but has failed, and he comes to us with the idea of having some operation made, which will relieve his condition.

I shall first find out the seat of the stricture and then attempt to pass an instrument into the bladder. Once through the stricture and into the bladder our way is clear, for if we can pass the smallest sized instrument another of larger calibre can be made to follow it and another still larger and so on until we are enabled to introduce the largest catheter. This you must not suppose is to be accomplished at once; it will, if we are successful, occupy weeks in its accomplishment. If we can introduce No. 2 to-day, No. 3 to-morrow, and three or four days after No. 4 or 5 we shall be accomplishing all we can wish to do. But I may fail in introducing any instrument whatever, or conditions may develope themselves which may indicate that this is not the best plan of treatment, what then remains to be done? At one time Liston, and Syme also, claimed that no stricture was impassable, and that any procedure which was based upon the theory that the stricture could not be penetrated was unnecessary. They subsequently withdrew these statements, and said that perineal section, or external urethrotomy, or external perineal urethrotomy, as it has been variously termed, might be found necessary in some instances.

This operation consists in cutting down upon the stricture dividing it and passing a catheter into the bladder. It is one of the difficult operations in surgery; although I have made it some five or six times successfully I expect sometime to meet a case which will tax all my resources. Unlike the operation of lithotomy it is accomplished in most instances without any guide, except the surgeon's anatomical knowledge, and the operator has to search through a mass of hardened urethral tissue for a canal too small in most cases to admit the passage of more than a drop of water at a time.

The instrument which I now show you is Thompson's dilator as improved by Dr. Gouley, of New York. He has so modified it that it can be used with these small whalebone guide bougies which I also show you. It is my intention to introduce one of these small whalebone bougies through the stricture if possible, and then attempt to pass one or more by its side; in this way I expect to be able to dilate the passage sufficiently to introduce a number one catheter, and by the use of gradual dilatation, introducing at intervals of three or four days catheters one or two numbers larger, to accomplish a cure.

I have now passed the small bougie through the first stricture and come upon a second one through which I find some difficulty in making a passage, and as our hour has expired I will excuse you for to-day after saying that every effort will be made to introduce one through the second stricture, and that you will be kept informed concerning the progress of the case.

Saturday, Oct. 18.—J. B. comes before you again to-day to report progress you will observe that he is now able to make water in a small stream whereas he could only pass it by drops when you first saw him. A short time after you first saw him, I passed a bougie into the bladder and by passsing another of the same size at its side have arrived at this result. Yesterday our patient had a chill and some fever which was not I think due, however, to the operation; it was controlled by quinine and to-day he is better.*

Wednesday, Oct. 15.—Mike B——, an Irishman, aged 46, comes before you to-day with the following history: Two years ago he first noticed pain and swelling in his left knee, the size of the knee gradually increased for six months accompanied with increased pain; at the end of that time he was obliged to take to his bed to which he has been confined for the past year and a half. He has what is commonly termed white swelling or scrofulous disease of the knee joint. With the characteristic preverseness of his race he

* Saturday, Oct. 25. Thompson's Dilator was introduced and opened to its full size and No. 14 Catheter introduced; the patient is doing well, no unpleasant consequences resulted.

has refused to allow anything to be done calculated to remove either the joint or the limb, declaring that death was preferable to amputation. When I saw him first at his home about a month since I detected a large accumulation of pus just above the knee which was causing him much pain and which I desired to evacuate, this he refused to have done, and you can now readily distinguish its seat both by sight and manipulation. After some persuasion he has consented to come into the Hospital and have his limb amputated, as the last resort. His disease has reached that stage that exsection if ever to be considered is now out of question, and amputation is attended with so many dangers that I am in great doubt of his surviving long after the operation.

In all amputations the mortality is as you know increased as the point of separation approaches the body. In this case while some few months ago we might have chosen the lower third as the seat of amputation now the tissues have been so undermined by the suppurative process that in order to get flaps to cover the bone we are obliged to amputate at the junction of the upper and middle thirds. In amputations of the thigh for injury statistics show that somewhat over one half recover, while in amputations performed for disease the mortality is a little less than twenty-five per cent.

In some cases the rapidity with which the patient will improve is almost remarkable. The loss of appetite, troublesome sweats and diarrhœa which have been so marked during the last stages of the disease will at once begin to abate, and the patient will be in every respect improved.

I propose to amputate in this instance by the flap method which I prefer to all others, forming the flaps from the anterior and posterior portions of the limb. As my knife enters the limb above the bone to form the anterior flap a large amount of pus escapes, this is however from the accumulation just above the joint and will not I think effect the flap as it is conveyed to this locality by a small sinus. I now cut outward and having made my first flap enter the knife immediately behind the bone and proceed in a like manner to make the second one which I desire to have a little longer than the first. The tourniquet does not seem to control the artery sufficiently, and before sawing off the bone I will take it up in these small spring forceps which affectually control the hemorrhage. After sawing off the bone I smooth off the rough edges with the bone forceps. Several arteries bleed which require ligature, and the flaps are then brought together by a few sutures and supported by bandages. His stump will be dressed with warm water, and he will receive stimulants and if necessary anodyne to relieve pain.

Wednesday, Oct. 22d.—Our patient is to-day greatly improved, he is more cheerful, does not have as much pain as before the operation, and declares that he has nothing to complain of except that he is not able to walk. We have thus far every reason to feel gratified at the result of the operation.*

* The diarrhœa, which was a troublesome complication of this case, was found difficult to check fully and he succumbed to its exhausting drain upon his already weakened system, early on the morning of October 29.

